# Oral health status of Dutch Armed Forces recruits in the years 2000, 2010 and 2020, a retrospective repeated cross-sectional study

**DOI:** 10.1186/s12903-024-04687-8

**Published:** 2024-08-08

**Authors:** A. J. de la Court, N. J. M. Opdam, E. M. Bronkhorst, M. Laske, M. C. D. N. J. M. Huysmans

**Affiliations:** Department of Dentistry, Radboud University Medical Center, Radboud Research Institute for Medical Innovation, Nijmegen, The Netherlands

**Keywords:** Oral health, Retrospective study, Socioeconomic status, Caries, Endodontic treatment, Dental trauma

## Abstract

**Background:**

Studies on oral health status of adults are sparse and rarely include data on endodontic treatment and trauma. In the military, those data are available because recruits are routinely assessed with a clinical and radiological examination at the start of their career. This study aimed to identify differences in oral health status of Dutch Armed Forces recruits between cohorts, departments, sex, age and rank, with DMF-T, endodontic treatment and dental trauma as outcome measures.

**Methods:**

Data from Electronic Patient Files from all recruits enlisted in 2000, 2010 and 2020 were used for analysis in a hurdle model resulting in the estimated cohort effect, controlled for the demographic variables. The total number of recruits was 5,764. Due to the retrospective character of the study a proxy was used to compose D-T and dental trauma.

**Results:**

The mean DMF-T number in recruits decreases from 5.3 in cohort 2000 to 4.13 in cohort 2010 and 3.41 in cohort 2020. The percentage of endodontically treated teeth increases from 6% in cohort 2000 to respectively 9% in 2010 and 8% in 2020. The percentage of recruits showing signs of dental trauma did not change significantly between cohort 2000 (3.1%) and cohort 2010 and 2020 (both 2.7%).

**Conclusions:**

Oral health in Armed Forces recruits is improving over the years, following a similar trend as the general population in the Netherlands. Lower SES represented by enlisted rank showed substantial lower oral health status.

## Background

Oral health is a substantial part of general health and wellbeing. Oral diseases are among the most prevalent diseases worldwide associated with considerable economic burden, decreased work productivity and reduced quality of life, in low-income countries as well as in industrialized countries [1–3]. Studies on oral health status of adults are rather sparse. In the Netherlands, a number of cross-sectional studies have been conducted in children and young adults aged 5-23 years and adults aged 25-74 years [4, 5]. They reported that oral health in the Netherlands is on average improving, but stagnating or even deteriorating for people with a lower socioeconomic status (SES).

Most of these studies only report on clinical measures like DMF-T. The DMF-T index is a standard method recommended by the World Health Organization to describe the amount of damage to the dentition of a person per tooth due to caries or, in other words, caries experience [6, 7]. Originally it is a clinical examination which is usually modified in the military by using bilateral bitewing radiographs and clinical assessment of recruits during their first visit to prevent underestimation of the need for restorative care [8]. Information on root canal treatment (RCT) in these cross-sectional studies is not included because they can only report on clinically collected data and are not able to derive information from patient files with radiographs needed to reliably asses the presence of endodontic treatment. Information on RCTs is valuable because these treatments are common and have an significant impact on oral health. Root filled teeth have a significantly greater risk for vertical root fracture due to a combination of loss of structural integrity, presence of pre-existing fractures and loss of vitality and is a common reason for tooth loss [9]. Traumatic dental injuries are common and mainly involve anterior teeth of the upper jaw [10, 11]. Their treatment is often complicated and can continue during the rest of a patient’s life especially in cases with more extensive damage to the hard dental tissues and the pulp, such as complicated crown fracture, uncomplicated crown- root fracture, complicated crown- root fracture, and root fractures [12]. Both endodontically treated teeth and dental trauma are indicative for substantial dental problems and pose a risk for pain and additional damage that requires extensive and costly care [11, 13]. It is important for policymakers to have insight into the expected demand for care in order to ensure sufficient capacity in healthcare.

In many countries such as Australia, Finland, Israel and New Zealand, the dental data of recruits are used to gain insight into the oral health status of young adults [14–17]. Between 2000-5000 young adults join the Dutch Ministry of Defense as recruits every year. Although this is a selected group, it is plausible it has certain representativity for young adults in general in the Netherlands. In the armed forces, this group is divided into ranks according to educational level, which may be an indicator for SES at this age [18–20]. During initial training, recruits are extensively mapped with both clinical and radiological examinations, resulting in a complete dental status, including clinically undetected (approximal) caries and endodontic treatments. Those data could be used for mapping the oral health status of young adults over several decades in a detailed way.

The aim of the present study was to investigate the oral health status of Dutch Armed Forces recruits including DMF-T, endodontic treatments and dental trauma, in 3 recruitment cohorts spanning 2 decades, and to identify differences in oral health status between cohorts, departments, sex, age and rank.

## Methods

This repeated cross-sectional study compares oral health of all military recruits of the Dutch Armed Forces enlisted in the years 2000, 2010 and 2020. The Armed Forces Dental Service electronic patient files were used to record oral health status with DMF-T, endodontic treatment and complicated dental trauma as outcome measures, and cohorts, departments, sex, age and rank as a proxy for SES as determinants.

The protocol of this study was rated by the local ethics committee as ‘no approval necessary’ (CMO Radboudumc file nr. 2019-5863).

The data were collected from the armed forces Electronic Patient Files (EPF) (Exquise software, Vertimart, Kwadijk, The Netherlands). Data of all patient files between 1999 and 2022 were extracted from the EPF using a script provided by the software supplier. Data were pseudonymized using the Statistical application ‘R’. To identify recruits, only patient numbers that first occurred in 2000, 2010 or 2020 were selected, resulting in 3453 files in 2000; 3470 in 2010 and 2292 in 2020. The following exclusion criteria were applied: Recruits younger than 18 and older than 30 years old; recruits with follow-up < 12 months; recruits with incomplete records.

### Outcome measures

#### DMF-T index

On enlistment, the dental history status of recruits was recorded by a dentist in the EPF including all previously restored and removed teeth. These teeth were defined as ‘Missing’ or ‘Filled’ (restored). Moreover, when a treatment need was established, this resulted in ‘planned procedures’. When the treatment was completed, the planned procedure was recorded as treatment in the EPF. Teeth with direct restorations and tooth extractions resulting from ‘planned procedures’ were recorded as decayed (D) on the day of entry. When a direct restoration replaced a previous restoration, the tooth with a ‘planned procedure’ was only recorded as filled (F). Third molars and their treatments were excluded from the analysis as they normally have a deviant clinical course.

As a result, components of the DMF-T index were assessed as follows:

1. Every new direct restoration in a tooth placed within 12 months after first entry without any recorded historic treatment code was considered decayed (D) at baseline.
2. Every tooth with a restoration code at first visit and an additional single surface being restored within 12 months, was considered to be decayed when the new restoration was not part of the existing filling.
3. Every mesial or distal surface filling added to an already existing filling within 12 months that not already had a mesial or distal filling was considered tooth decay (D) at baseline.
4. Every missing tooth at entry (except third molars) were considered as missing (M) at baseline
5. All restored teeth at entry, without any follow-up treatment within 12 months were considered as Filled (F)

### Presence of Root Canal Treatment (RCT)

All teeth containing root canal treatment observed during the radiographic examination at first entry were recorded.

### Complicated dental trauma

In this study we considered a recruit to have suffered complicated dental trauma when one or more front teeth (upper and lower, cuspids included) were missing, endodontically treated, and/or provided with a crown. It should be noted that this is used as a proxy for damage caused by traumatic dental injuries. The presence of direct restorations in front teeth was not considered as a sign for dental trauma.

### Statistical analysis

The analysis aimed to estimate differences in oral health status between cohorts. As cohorts differed over time regarding age, sex, rank and department these variables were included in our analyses, resulting in the estimated cohort effect, controlled for the demographic variables. In our study, military rank is used as substitute for socio-economic status (SES), where we consider enlisted ranks as ‘low’ and cadets (officer trainees / potential officer ranks) as ‘high’ SES as only recruits having received a higher-level secondary or tertiary education can qualify as officer [20].

For the analysis R version 4.05 was used. All outcomes can be seen as count data, with a (very) high prevalence of count of 0. Therefore, hurdle models were used to analyze the relation between properties of recruits and the various counts. Hurdle models consist of two parts, one estimating the occurrence of either a count of 0 or a larger count and the other part is a truncated count negative binomial model that models the positive counts. This resulted in Odds Ratios (OR) that show the association between two factors, and Incidence Risk Ratios (IRR) that show association with the actual count, if larger than 0. For example, the OR in our study describes the odds for having a DMF-T > 0 between one cohort compared to the other, the IRR describes the relative amount of DMF-T and can be considered as severity of DMF-T.

## Results

### Study population

The entire number of recruits in 2000, 2010 and 2020 was 9215. 3451 recruits were excluded; 497 because they were younger than 18 or older than 30 years old; 1919 due to a follow-up < 12 months; 1035 due to incomplete records. Therefore, the total number of recruits available for analysis in this study was 5,764: 2,203 in 2000; 2,130 in 2010 and 1,431 in 2020. The number of female recruits differed between cohorts: 16,6% in 2000; 11,2% in 2010 and 19,6% in 2020. The fraction of cadets (officers in training) was about 10% of all recruits (2000: 6,4%; 2010: 10,1%; 2020 12,2%) (Table 1).

**Table 1:** Characteristics of the Royal Netherlands Armed Forces recruits included in this study, by cohort

	**2000 (*n* = 2203)**	**2010 (*n* = 2130)**	**2020(*n* = 1431)**
**Age in years, mean (SD)**	20.40 (2.44)	20.86 (2.84)	22.00 (3.08)
**Sex, n (%)**			
** Male**	1838 (83.40)	1891 (88.80)	1150 (80.40)
** Female**	365 (16.60)	239 (11.20)	281 (19.60)
**Rank, n (%)**			
** Cadet**	140 (6.40)	216 (10.10)	174 (12.20)
** Enlisted**	2063 (93.60)	1914 (89.90)	1257 (87.80)
** Army**			
** n (%)**	1340 (60.80)	1356 (63.70)	894 (62.50)
** Age in years, mean (SD)**	20.24 (2.37)	20.70 (2.37)	21.70 (2.90)
**Airforce**			
** n (%)**	357 (16.20)	300 (14.10)	282 (19.70)
** Age in years, mean (SD)**	21.31 (2.71)	21.76 (3.07)	23.22 (3.41)
**Navy**			
** n (%)**	506 (23.00)	474 (22.30)	255 (17.80)
** Age in years, mean (SD)**	20.17 (2.29)	20.74 (2.66)	21.70 (3.00)

### DMF-T and separate D-T, M-T, and F-T

**Fig. 1:**
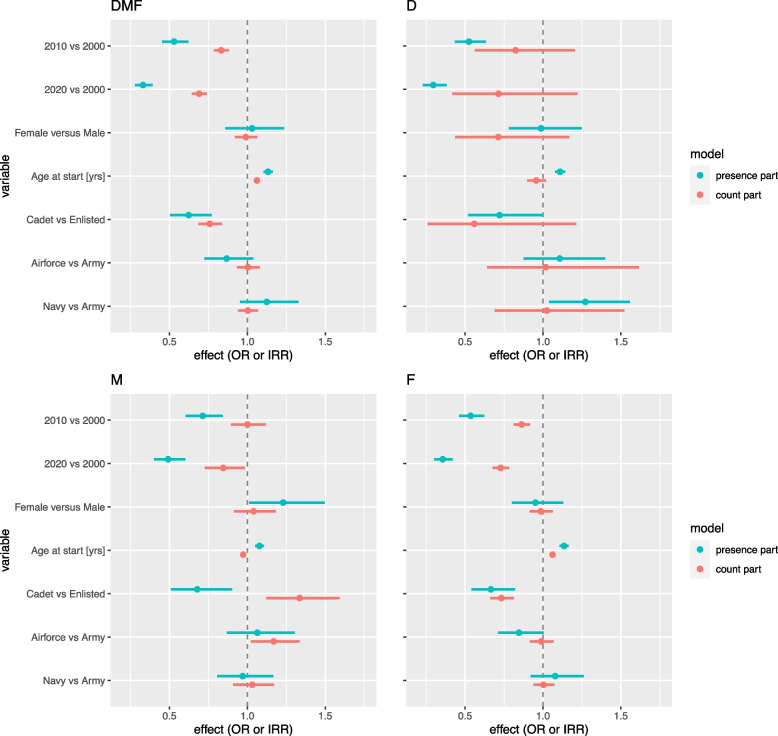
Hurdle model analyses for DMF and separate D, M, and F. Hurdle model analyses comparing cohort 2010 and 2020 with 2000, females with males, age, cadets versus enlisted rank, Navy and Air Force with Army. The presence part displays the Odds Ratios (OR) and is indicated by the blue line. The count part displays the Incidence Risk Ratios (IRR) and is indicated by the red line. In both OR and IRR the width of the line represents the 95% confidence interval

Descriptives are shown in Table 2. Although D,M, and F together are a measure for caries experience, they have different impact on oral health and can point out differences in level of dental care and treatment need. Therefore, we analyzed both DMF-T and D-T, M-T, and F-T, separately. Analyses are shown in Fig. 1, associated IRR, OR with P value and 95% confidence interval are shown in Table 3.

**Table 2:** Number, percentage, mean and maximum value of DMF-T, teeth with root canal treatment (RCT), and with complicated dental trauma (CDT) derived from RCT, indirect restorations (crowns), and Missing, by cohort

	**0 n (%)**	**1 n (%)**	**2 n (%)**	**3 n (%)**	**4 n (%)**	**5 n (%)**	≥**6 n (%)**	**Mean (SD)**	**max**
**2000** ***n* = 2203**									
**DMF-T**	311 (14.1)	222 (10.1)	211 (9.6)	193 (8.8)	210 (9.5)	158 (7.2)	898 (40.8)	5.30 (4.5)	23
**Decayed**	1860 (84.4)	229 (10.4)	64 (2.9)	36 (1.6)	9 (0.4)	3 (0.1)	2 (0.1)	0.24 (0.7)	10
**Missing, total**	1803 (81.8)	111 (5.0)	120 (5.4)	20 (0.9)	136 (6.2)	4 (0.2)	9 (0.4)	0.47 (1.2)	8
**Missing, anterior**	2165 (98,3)	24 (1,1)	14 (0,6)	0 (0.0)	0 (0.0)	0 (0.0)	0 (0.0)	0.02 (0.2)	2
**Missing, premolars**	1892 (85,9)	66 (3)	101 (4,6)	16 (0,7)	123 (5,6)	2 (0,1)	2 (0,1)	0.38 (1.05)	8
**Filled**	367 (16.7)	247 (11.2)	226 (10.3)	220 (10.0)	208 (9.4)	175 (7.9)	760 (34.5)	4.59 (4.1)	22
**RCT, anterior**	2159 (98.0)	37 (1.7)	7 (0.3)	0 (0.0)	0 (0.0)	0 (0.0)	0 (0.0)	0.02 (0.2)	2
**RCT, posterior**	2111 (95.8)	79 (3.6)	12 (0.5)	0 (0.0)	0 (0.0)	1 (0.0)	0 (0.0)	0.05 (0.3)	5
**Crowns, anterior**	2157 (97.9)	27 (1.2)	10 (0.5)	3 (0.1)	4 (0.2)	0 (0.0)	2 (0.1)	0.04 (0.3)	7
**CDT**	2134 (96.9)	40 (1.8)	21 (1.0)	4 (0.2)	2 (0.1)	0 (0.0)	2 (0.1)	0.05 (0.3)	6
**2010 *n* = 2130**									
** DMF-T**	489 (23.0)	283 (13.3)	235 (11.0)	166 (7.8)	189 (8.9)	132 (6.2)	636 (29.9)	4.13 (4.3)	25
** Decayed**	1934 (90.8)	139 (6.5)	42 (2.0)	8 (0.4)	5 (0.2)	1 (0.0)	2 (0.1)	0.13 (0.5)	9
** Missing, total**	1836 (86.2)	78 (3.7)	97 (4.6)	10 (0.5)	107 (5.0)	1 (0.0)	1 (0.0)	0.35 (1.0)	6
** Missing, anterior**	2107 (98,9)	13 (0,6)	9 (0,4)	1 (0)	0 (0.0)	0 (0.0)	0 (0.0)	0.02 (0.2)	2
** Missing, premolars**	1914 (89,9)	37 (1,7)	81 (3,8)	5 (0,2)	92 (4,3)	1 (0)	0 (0)	0.28 (0.90)	5
** Filled**	558 (26.2)	302 (14.2)	233 (10.9)	178 (8.4)	160 (7.5)	139 (6.5)	560 (26.3)	3.65 (3.9)	25
** RCT, anterior**	2056 (96.5)	61 (2.9)	11 (0.5)	2 (0.1)	0 (0.0)	0 (0.0)	0 (0.0)	0.04 (0.2)	3
** RCT, posterior**	1994 (93.6)	110 (5.2)	21 (1.0)	2 (0.1)	2 (0.1)	1 (0.0)	0 (0.0)	0.08 (0.3)	5
** Crowns, anterior**	2109 (99.0)	13 (0.6)	7 (0.3)	1 (0.0)	0 (0.0)	0 (0.0)	0 (0.0)	0.01 (0.2)	3
** CDT**	2072 (97.3)	41 (1.9)	14 (0.7)	2 (0.1)	1 (0.0)	0 (0.0)	0 (0.0)	0.04 (0.2)	4
**2020 *n* = 1431**									
** DMF-T**	424 (29.6)	199 (13.9)	166 (11.6)	105 (7.3)	122 (8.5)	88 (6.1)	327 (22.9)	3.41 (4.1)	28
** Decayed**	1345 (94.0)	70 (4.9)	10 (0.7)	1 (0.1)	2 (0.1)	2 (0.1)	1 (0.1)	0.08 (0.4)	6
** Missing, total**	1275 (89.1)	60 (4.2)	45 (3.1)	6 (0.4)	42 (2.9)	2 (0.1)	1 (0.1)	0.25 (0.8)	6
** Missing, anterior**	1414 (98,8)	13 (0,9)	3 (0,2)	0 (0.0)	1 (0,1)	0 (0.0)	0 (0.0)	0.02 (0.2)	4
** Missing, premolars**	1334 (93,2)	24 (1,7)	31 (2,2)	6 (0,4)	36 (2,5)	0 (0)	0 (0)	0.17 (0.72)	4
** Filled**	459 (32.1)	214 (15.0)	168 (11.7)	114 (8.0)	101 (7.1)	87 (6.1)	288 (20.1)	3.08 (3.8)	28
** RCT, anterior**	1386 (96.9)	30 (2.1)	13 (0.9)	1 (0.1)	0 (0.0)	0 (0.0)	1 (0.1)	0.05 (0.3)	9
** RCT, posterior**	1352 (94.5)	62 (4.3)	11 (0.8)	4 (0.3)	1 (0.1)	0 (0.0)	1 (0.1)	0.07 (0.4)	6
** Crowns, anterior**	1421 (99.3)	5 (0.3)	4 (0.3)	0 (0.0)	0 (0.0)	0 (0.0)	1 (0.1)	0.01 (0.2)	8
** CDT**	1392 (97.3)	24 (1.7)	14 (1.0)	0 (0.0)	1 (0.1)	0 (0.0)	0 (0.0)	0.04 (0.3)	4

**Table 3:** IRR, OR with *P* value and 95% confidence interval

**DMF-T**	**OR**	**95% CI**	***P* value**	**IRR**	**95% CI**	***P* value**
**2010 vs 2000**	0.53	[0.45...0.62]	<0.001	0.83	[0.78...0.88]	<0.001
**2020 vs 2000**	0.33	[0.28...0.39]	<0.001	0.69	[0.64...0.74]	<0.001
**Female vs Male**	1.03	[0.86...1.24]	0.745	0.99	[0.92...1.06]	0.778
**Age at start**	1.13	[1.10...1.16]	<0.001	1.06	[1.05...1.07]	<0.001
**Cadets vs Enlisted**	0.62	[0.50...0.77]	<0.001	0.76	[0.69...0.84]	<0.001
**Airforce vs Army**	0.87	[0.72...1.04]	0.121	1.00	[0.93...1.08]	0.930
**Navy vs Army**	1.13	[0.95...1.33]	0.166	1.00	[0.94...1.07]	0.919
**D-T**						
** 2010 vs 2000**	0.53	[0.43...0.64]	<0.001	0.82	[0.56...1.21]	0.320
** 2020 vs 2000**	0.30	[0.23...0.38]	<0.001	0.71	[0.42...1.22]	0.220
** Female vs Male**	0.99	[0.78...1.25]	0.915	0.71	[0.44...1.17]	0.180
** Age at start**	1.11	[1.08...1.14]	<0.001	0.96	[0.90...1.02]	0.170
** Cadets vs Enlisted**	0.72	[0.52...1.00]	0.051	0.56	[0.26...1.21]	0.140
** Airforce vs Army**	1.11	[0.88...1.40]	0.392	1.02	[0.64...1.62]	0.940
** Navy vs Army**	1.27	[1.04...1.56]	0.020	1.03	[0.69...1.52]	0.900
**M-T**						
** 2010 vs 2000**	0.71	[0.60...0.84]	<0.001	1.00	[0.90...1.12]	1.000
** 2020 vs 2000**	0.49	[0.40...0.60]	<0.001	0.85	[0.73...0.98]	0.030
** Female vs Male**	1.23	[1.01...1.50]	0.038	1.04	[0.91...1.18]	0.550
** Age at start**	1.08	[1.05...1.11]	<0.001	0.97	[0.96...0.99]	<0.001
** Cadets vs Enlisted**	0.68	[0.51...0.90]	0.008	1.34	[1.12...1.59]	<0.001
** Airforce vs Army**	1.06	[0.87...1.30]	0.551	1.17	[1.02...1.34]	0.020
** Navy vs Army**	0.97	[0.81...1.17]	0.746	1.03	[0.91...1.17]	0.630
**M anterior**						
** 2010 vs 2000**	0.58	[0.34…0.99]	0.045	1.40	[0.63…3.12]	0.411
** 2020 vs 2000**	0.58	[0.32…1.06]	0.076	1.08	[0.41…2.88]	0.875
** Female vs Male**	0.84	[0.44…1.61]	0.599	1.26	[0.43…3.71]	0.671
** Age at start**	1.10	[1.02…1.18]	0.016	0.95	[0.84…1.07]	0.383
** Cadets vs Enlisted**	1.05	[0.49…2.25]	0.906	0.88	[0.28…2.76]	0.825
** Airforce vs Army**	1.00	[0.54…1.85]	0.994	0.66	[0.23…1.86]	0.426
** Navy vs Army**	1.04	[0.60…1.83]	0.883	0.48	[0.17…1.40]	0.181
**F-T**						
** 2010 vs 2000**	0.54	[0.46...0.62]	<0.001	0.86	[0.81...0.92]	<0.001
** 2020 vs 2000**	0.36	[0.30…0.42]	<0.001	0.73	[0.68...0.78]	<0.001
** Female vs Male**	0.95	[0.80…1.13]	0.572	0.99	[0.91…1.06]	0.710
** Age at start**	1.14	[1.11...1.16]	<0.001	1.06	[1.05…1.07]	<0.001
** Cadets vs Enlisted**	0.67	[0.54...0.82]	<0.001	0.73	[0.66...0.81]	<0.001
** Airforce vs Army**	0.85	[0.71...1.01]	0.058	0.99	[0.92...1.07]	0.780
** Navy vs Army**	1.08	[0.92...1.26]	0.350	1.00	[0.94...1.07]	0.920
**F anterior**						
** 2010 vs 2000**	0.82	[0.71…0.94]	0.005	1.55	[1.26…1.89]	<0.001
** 2020 vs 2000**	0.65	[0.55…0.77]	<0.001	1.18	[0.92…1.50]	0.195
** Female vs Male**	0.83	[0.69…0.99]	0.037	1.12	[0.86…1.45]	0.392
** Age at start**	1.07	[1.05…1.10]	<0.001	1.03	[1.00…1.07]	0.040
** Cadets vs Enlisted**	0.59	[0.47…0.75]	<0.001	0.94	[0.65…1.36]	0.741
** Airforce vs Army**	1.06	[0.89…1.26]	0.500	0.83	[0.64…1.07]	0.143
** Navy vs Army**	0.97	[0.83…1.13]	0.673	0.80	[0.64…1.00]	0.052
**Endo posterior**						
** 2010 vs 2000**	1.46	[1.11...1.93]	0.007	1.97	[0.90...4.31]	0.090
** 2020 vs 2000**	1.08	[0.79...1.49]	0.637	2.18	[0.94...5.05]	0.070
** Female vs Male**	0.97	[0.70...1.35]	0.855	0.91	[0.39...2.11]	0.820
** Age at start**	1.18	[1.13...1.22]	<0.001	1.09	[0.99...1.19]	0.090
** Cadets vs Enlisted**	0.45	[0.28...0.75]	0.002	1.17	[0.33...4.19]	0.810
** Airforce vs Army**	0.77	[0.54...1.09]	0.145	0.80	[0.31...2.08]	0.640
** Navy vs Army**	1.33	[1.01...1.74]	0.040	2.24	[1.13...4.45]	0.020
** Endo anterior**						
** 2010 vs 2000**	1.76	[1.20...2.57]	0.004	1.07	[0.42...2.72]	0.890
** 2020 vs 2000**	1.64	[1.07...2.53]	0.025	1.91	[0.72...5.02]	0.190
** Female vs Male**	0.76	[0.47...1.24]	0.278	1.21	[0.47...3.08]	0.700
** Age at start**	1.03	[0.97...1.09]	0.308	1.11	[0.99...1.24]	0.080
** Cadets vs Enlisted**	0.44	[0.20...0.95]	0.036	4.69	[2.02...10.85]	<0.001
** Airforce vs Army**	0.8	[0.48...1.32]	0.376	0.33	[0.10...1.01]	0.050
** Navy vs Army**	1.52	[1.07...2.16]	0.020	0.73	[0.34...1.59]	0.430
**Crowns anterior teeth**						
** 2010 vs 2000**	0.42	[0.25…0.71]	0.001	0.49	[0.20…1.21]	0.120
** 2020 vs 2000**	0.23	[0.11…0.46]	<0.001	1.07	[0.35…3.22]	0.905
** Female vs Male**	0.74	[0.38…1.46]	0.387	1.91	[0.76…4.81]	0.170
** Age at start**	1.23	[1.14…1.32]	<0.001	1.04	[0.91…1.20]	0.533
** Cadets vs Enlisted**	0.39	[0.14…1.09]	0.073	0.46	[0.05…4.72]	0.517
** Airforce vs Army**	1.33	[0.78…2.29]	0.295	0.45	[0.17…1.18]	0.104
** Navy vs Army**	0.50	[0.24…1.03]	0.059	1.69	[0.59…4.88]	0.328
**CDT (anterior Crowns, E or M)**						
** 2010 vs 2000**	0.84	[0.58…1.19]	0.324	0.54	[0.32…0.92]	0.024
** 2020 vs 2000**	0.81	[0.54…1.22]	0.313	0.62	[0.34…1.11]	0.108
** Female vs Male**	0.70	[0.43…1.13]	0.146	1.47	[0.80…2.71]	0.214
** Age at start**	1.06	[1.00…1.11]	0.052	1.10	[1.02…1.19]	0.016
** Cadets vs Enlisted**	0.73	[0.40…1.35]	0.316	1.15	[0.51…2.57]	0.737
** Airforce vs Army**	0.94	[0.61…1.46]	0.798	0.62	[0.32…1.19]	0.149
** Navy vs Army**	0.92	[0.62…1.37]	0.694	0.60	[0.31…1.17]	0.134

The analysis shows that caries experience in cohorts declined from mean DMF-T 5.30 (SD 4.5) in 2000 to 4.13 (SD 4.3) in 2010 to 3.41 (SD 4.1) in 2020. Between 2000 and 2020, the proportion of recruits with a sound dentition increased and the DMF-T score of recruits with caries experience decreased, as can be seen from the OR and IRR being clearly smaller than 1. No differences in DMF-T were found for sex. As expected the numbers are higher with increasing age.

In our hurdle model (see Fig. 1) we compared the outcome measures of recruits to each other in terms of odds ratio (OR) and incidence rate ratio (IRR): cohort 2010 and 2020 were compared with cohort 2000; for SES cadets with enlisted; female with male; Navy and Air Force with Aarmy. The separate analyses for DMF showed that the Filled part is the most decisive factor for caries experience in recruits. Missing and Decayed are less frequent, but show some noteworthy results. The number of missing teeth in cadets is higher although the number of cadets with missing teeth is lower compared to enlisted recruits. Males and females show a comparable fraction of recruits with caries, but females with caries show a lower number of cavities, although with a wide margin of uncertainty.

### Root canal treatment

**Fig. 2:**
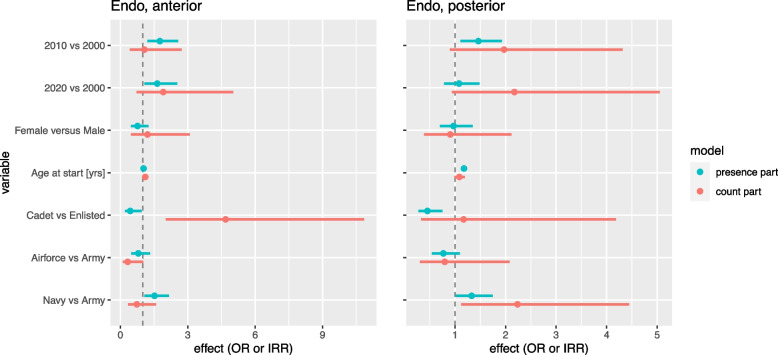
Hurdle model analyses for endodontic treatment separated for anterior and posterior teeth. Hurdle model analyses comparing cohort 2010 and 2020 with 2000, females with males, age, cadets versus enlisted rank, Navy and Air Force with Army. The presence part displays the Odds Ratios (OR) and is indicated by the blue line. The count part displays the Incidence Risk Ratios (IRR) and is indicated by the red line. In both OR and IRR the width of the line represents the 95% confidence interval

The number and percentage of root-filled teeth can be found in Table 2. The results of the statistical analyses are shown in Fig. 2, associated IRR, OR with P value and 95% confidence interval are shown in Table 3. The number of endodontic treated teeth in recruits was limited. In 2000 6% of recruits had one or more endodontically treated teeth. In 2010 it increased to 9% and in 2020 8% of recruits had root-filled teeth.

Both cohort 2010 and 2020 have a higher share of recruits with endodontic treatment in anterior teeth than the 2000 cohort, although the number of endodontically treated teeth in these recruits is comparable in 2010 and somewhat higher in 2020. More recruits in 2010 have endodontically treated posterior teeth but in cohort 2020 it is similar to cohort 2000. However, in cohort 2010 and 2020 most recruits with endodontic treatment have a higher number of root-filled teeth. A slightly lower number of cadets have experienced endodontic treatment but those who have, show higher numbers of root-filled teeth, especially in front teeth.

### Complicated dental trauma

**Fig. 3:**
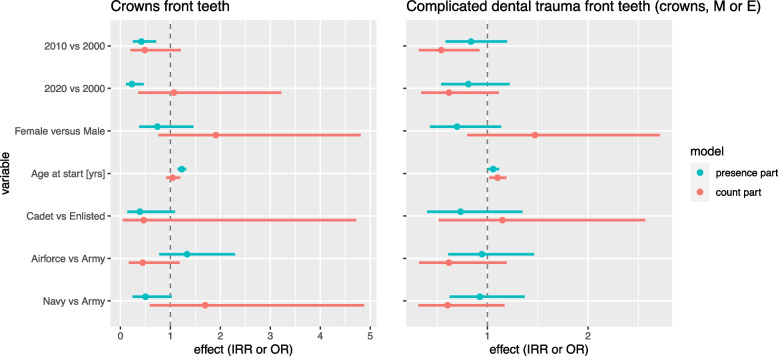
Hurdle model analyses for crowns on anterior teeth and complicated dental trauma. Hurdle model analyses comparing cohort 2010 and 2020 with 2000, females with males, age, cadets versus enlisted rank, Navy and Air Force with Army. The presence part displays the Odds Ratios (OR) and is indicated by the blue line. The count part displays the Incidence Risk Ratios (IRR) and is indicated by the red line. In both OR and IRR the width of the line represents the 95% confidence interval

**Fig. 4:**
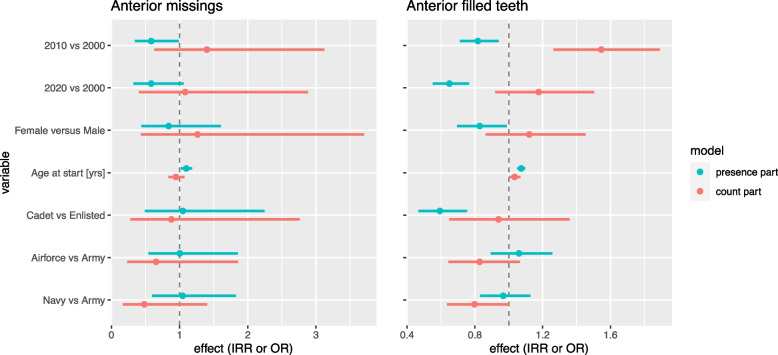
Hurdle model analyses for anterior missing teeth and anterior filled teeth. Hurdle model analyses comparing cohort 2010 and 2020 with 2000, females with males, age, cadets versus enlisted rank, Nnavy and Air Force with Army. The presence part displays the Odds Ratios (OR) and is indicated by the blue line. The count part displays the Incidence Risk Ratios (IRR) and is indicated by the red line. In both OR and IRR the width of the line represents the 95% confidence interval

The number and percentage of complicated dental trauma in anterior teeth as a proxy for traumatic dental injuries can be found in Table 2. The results of the statistical analysis are shown in Figs. 3 and 4, associated IRR, OR with P value and 95% confidence interval are shown in Table 3. Using endodontic treatment, indirect restorations and missing teeth in the anterior region as an indication for complicated dental trauma, the prevalence was 3.1% in cohort 2000; 2.7% in cohort 2010 and 2.7% in cohort 2020. The number of missing anterior teeth shows a modest decrease, while the number of endodontically treated anterior teeth shows an increase. Fewer female recruits have signs of dental trauma, but the ones that have, tend to have more damaged teeth, but with a wide distribution. In cadets we see a similar effect, but less pronounced. Age shows limited influence on the prevalence of dental trauma.

## Discussion

This study shows that the oral health status of Dutch Armed Forces recruits in general has improved over the last two decades. DMF-T score shows a significant decrease from cohort 2000 compared to cohort 2010 and cohort 2020, and the percentage of recruits with a sound dentition increases significantly. Significant increases were also found in endodontic treatment. Traumatic Dental Injuries show no significant differences between the three cohorts.

This outcome of armed forces recruits may also be relevant for Dutch young adults in the general population as this is the first cross-sectional study in the Netherlands based on such a large quantity of data on developments in oral health in young adults with radiographic controlled findings. Royal Netherlands Armed Forces Dental Service provides a unique collection of oral health data, because it is obliged to keep up-to-date dental records of all military personnel with recent radiographs for forensic reasons. Therefore, Dutch Armed Forces data combine clinical and radiographic findings of all recruits and contains detailed information on missing, filled and decayed teeth. In most epidemiological studies this is not possible, because taking radiographs for research purposes only is considered unethical [21].

The present study also provides detailed information about endodontic treatment for a large population sample. This is valuable because such studies are limited to one study in the Netherlands [22] and a French study [23] showing a very wide range of endodontic treatment percentages in different populations.

With data on clinical and radiological findings combined, it is possible to reconstruct the more prominent (severe) damage caused by traumatic dental injuries. Although it is a reconstruction, we consider it valuable because information on numbers of dental trauma is sparse, both in the Netherlands as worldwide [10].

The fact that our epidemiological survey is limited to Armed Forces recruits has limitations as well as advantages. Our study only describes oral health status of young adults selected for the military. This selected group has a much lower percentage of females and a different distribution of high and low SES compared to the general population. Although the military provides a clear distinction between high and low SES on basis of educational level in the same way cross-sectional studies as ‘Kies voor Tanden’[4] in the Netherlands uses, the distribution of higher and lower SES is different. It is probable that the lower SES military differs in general health and economic status because they were all medically assessed and have a paid occupation, which is not always the case in the lower SES population in the Netherlands in general. The results of this study should be interpreted carefully when compared to Dutch young adults. Furthermore, patient factors such as oral hygiene and smoking were collected during oral examination, but these data were not retrievable in a standardized way from the electronic patient files. Therefore, the study has a lack of certain patient characteristics. On the other hand, the investigated cohorts can be considered as young, healthy adults and are examined thoroughly and in a standardized protocol by a military dentist.

The total size of cohorts 2000 and 2010 are similar. Cohort 2020 is about a third smaller because in the first months of 2020 there were no recruits to the armed forces due to COVID-19 regulations. However, we did not expect the proportions of oral health aspects would be different in this cohort.

Due to the retrospective character of these data, the clinical information required for this study was not (directly) available for all different categories of traumatic dental injuries or for caries (D). However, the available data provides information on the treatment most probably followed by traumatic dental injuries and diagnosed caries lesions. Therefore, information on crowns, root canal treatment and M-T in anterior teeth was combined as a proxy for complicated dental trauma. Minor trauma will not be reflected in this outcome measure. However, the more severe nature of these traumatic injuries has greater need for follow-up treatment, which would be more meaningful for policy makers.

It should also be noted that in this study the D-component is derived from performed procedures in the first 12 months after enlistment. This may lead to both an overestimation and an underestimation of D-T. Overestimation, because restorations placed due to fractures (e.g., cusp replacements) may have been counted as D-T, and underestimation as replacements due to secondary caries in the same surfaces were not counted as D-T. Secondary caries and fracture are considered the most common reason for restoration failure [24, 25]. The combined effect of mis-categorization is considered to be limited, because the D-prevalence is low and only a modest part of DMF-T score.

Our data indicate that DMF-T decreases over time for the cohorts, both in fraction of recruits with caries experience (DMF-T>0) as in the DMF-T score per individual. As expected, caries experience among recruits increases with age because the effect of DMF-T score is cumulative.

These findings reflect the trend of decreasing DMF-T in high-income countries worldwide [26–28] and in the Dutch National Oral Health Surveys [4, 5]. DMF-T and the proportions of separate D, M, and F in the Dutch National Oral Health Surveys show a downward trend of children and young adults in all age groups. When compared with the national survey data, the numbers of the 23-year-olds in this report with our recruits (mean age 20.40 – 22.00) show a mostly similar trend. Only their last finding from 2017 for high SES 23-year-olds shows a slightly higher caries experience and DMF-T compared to 2011, a stagnation in the diminishing caries experience that our data are not showing.

When comparing separate components, recruits show a lower number of D-T but a higher number of M-T. A possible explanation is that recruits are encouraged to be fit before enlisting for the armed force and visit their home dentist before starting their military career.

Our data do not differentiate between Missing due to caries or Missing due to other reasons like orthodontic treatment, which is, third molars not taking into account, the main reason for tooth extraction in young adults [29]. To prevent or correct malocclusion, it is most common to extract premolars instead of other types of teeth and missing accounts. In all three cohorts a substantial part of recruits has missing premolars. It is probable this is for orthodontic reasons and not due to caries.

The rate of cadets (officers in training) with caries experience is significantly lower compared to enlisted personnel; their DMF-T score is also lower. This is in line with an earlier study on health disparities in the Dutch Armed Forces [20]. Rank can be considered as educational level which is used as a proxy for SES in several other studies as SES has been documented to impact the ability to acquire and interpret health information [19, 30, 31]. The educational level is also associated to social position, having a higher level of social support and having access to dental care, so it might explain that recruits with higher SES and rank show lower risks and have less oral health problems. In our study, we also assessed SES based on zip code in our analysis but this proved not to be a significant factor probably because the scale local governments use to register SES is not detailed enough. Although females tend to have a slightly higher rate of tooth decay [32], in our study male and female recruits showed a comparable caries prevalence.

In contrast to the findings on caries experience, the number of endodontically treated teeth increases over time. Specifically, the number of endodontic treatment performed in the posterior region is much higher in cohort 2010 (OR 1.76) although the effect is less pronounced in cohort 2020 (OR 1.64). Posterior region endodontic treatment is mostly related to deep cavities or large fillings due to caries. In populations with more caries experience it is likely they show higher numbers of RCT, for example in the Navy both the proportion of Decayed (D) and of endodontically treated teeth (E) in the posterior region are higher compared to the Army (D Navy OR 1.27; E post Navy OR 1.33). At the same time the number of missing teeth (M) decreased in cohorts 2010 and 2020. The same effect is visible in the number of missing teeth in Navy personnel. These findings suggest that more teeth receive endodontic treatment instead of being extracted in recent years and is considered a sign of improved level of care. In 2006 health insurance was reformed in the Netherlands resulting in complete dental care available for people younger than 18 years old [33]. Around this time a publication on trends in oral healthcare reported a higher incidence of untreated cavities in children and adolescents [34]. As a consequence, oral health preventive programs were carried out. However, this effect is only significant in 2010 while cohort 2020 resonates the findings from 2000. Comparing these numbers and proportions to other populations is difficult because epidemiological data on prevalence of endodontic treatment are sparse and reports are mostly on specific and very diverse groups [13, 35, 36].

The proportion of endodontically treated anterior teeth also increased in the more recent cohorts. Endodontic treatment in anterior teeth is mostly carried out after trauma and only rarely after treating deep caries lesions. Root filled teeth in the anterior region are considered to be most related to traumatic dental injuries, while endodontically treated teeth in the posterior region are considered to be more related to caries [12, 37].

Complicated dental trauma includes traumatic dental injuries like complicated tooth fractures, luxations and avulsions and is likely to result in endodontic treatment, large restorations such as crowns, and missing teeth to be replaced. Although the proportion of recruits with endodontic treatment in anterior teeth increases over the years, complicated dental trauma tends to remain comparable among the three cohorts (cohort 2010 (mean 0.04; SD 0.2) and 2020 (mean 0.04; SD 0.3) compared to 2000 (mean 0.05; SD 0.3). At the same time Missing anterior teeth show a small decrease when cohort 2010 and 2020 are compared to cohort 2000 (2010: OR 0.58, CI 0.34 – 0.99; 2020: OR 0.58, CI 0.32 – 1.06). In 2010 and 2020 a lower percentage of recruits were treated with crowns on anterior teeth. At the same time, an increase in anterior filled teeth was expected, but our numbers for filled anterior teeth also decreases. It seems the number of trauma is relatively stable but the chosen treatment changes over time.

Other studies to which our trauma results can be compared are sparse. A meta-analysis from Petti et all [10] provides data from nations worldwide including European nations including all reported TDI’s in several regions, different populations and ages in both primary as well as permanent dentition. For European region their study reports a 14.0% prevalence, median age of 13.2 and proportion male-to-female prevalence ratio 1.48. In our study prevalence is much lower (3.1% in cohort 2000; 2.7% in cohort 2010 and 2020) because our study only reports the more severe levels of trauma. The male-to-female prevalence ratio we found (1.43) is very close to the 1.48 Pettis et al reports.

The only study in the Netherlands that reports on dental trauma is the ‘Kies voor tanden’ study [4]. It reports on missing anterior teeth due to trauma 0.9% in 2012 and 0.0% in 2017, and on crowns on anterior teeth due to trauma 1.2% in 2012 and 0.6% in 2017 for 23-year-olds. Our study shows the same trend of decreasing missing teeth and anterior crowns although in other cohorts and age range.

## Conclusions

In conclusion, this study demonstrated that in Armed Forces recruits the oral health is improving over the years, with 14.1% of recruits having a sound dentition in 2000 and 29.6 % in 2020, following a similar trend as the general population in the Netherlands. The number of endodontically treated teeth increased, likely related to an improved level of dental care in recent years, and less tooth extractions. Male and female recruits show comparable oral health status. Lower rank (enlisted) showed substantial lower oral health status in all three outcome measures compared to higher rank (cadets) indicating that SES has substantial influence on oral health status, in this young adult population.

## Data Availability

The data that support the findings of this study are available from Health Care Department of the Ministry of Defense but restrictions apply to the availability of these data, which were used under license for the current study, and so are not publicly available. Data are however available from the authors upon reasonable request and with permission of Health Care Department of the Ministry of Defense.

## Abbreviations

DMF-T: Decayed, missing, filled, teeth
E: Endodontic treatment
SES: Socioeconomic status
EPF: Electronic patient files
RCT: Root canal treatment
CDT: Complicated dental trauma
OR: Odds ratios
IRR: Incidence rate ratios
SD: Standard deviation
CI: Confidence interval
Rest: Restoration
